# Green Synthesis of Silver Nanoparticles Incorporated Aromatherapies Utilized for Their Antioxidant and Antimicrobial Activities against Some Clinical Bacterial Isolates

**DOI:** 10.1155/2022/2432758

**Published:** 2022-04-11

**Authors:** Ahmed A. H. Abdellatif, Sultan S. Alhathloul, Abdullah S. M. Aljohani, Hamzah Maswadeh, Emad M. Abdallah, Khalid Hamid Musa, Mohamed A. El Hamd

**Affiliations:** ^1^Department of Pharmaceutics, College of Pharmacy, Qassim University, Buraydah 51452, Saudi Arabia; ^2^Department of Pharmaceutics and Pharmaceutical Technology, Faculty of Pharmacy, Al-Azhar University, Assiut 71524, Egypt; ^3^Department of Veterinary Medicine, College of Agriculture and Veterinary Medicine, Qassim University, Buraydah, Saudi Arabia; ^4^Department of Science Laboratories, College of Science and Arts, Qassim University, Ar Rass 51921, Saudi Arabia; ^5^Department of Food Science and Human Nutrition, College of Agriculture and Veterinary Medicine, Qassim University, Buraydah, Saudi Arabia; ^6^Department of Pharmaceutical Sciences, College of Pharmacy, Shaqra University, Al Dawadmi 11961, Saudi Arabia; ^7^Department of Pharmaceutical Analytical Chemistry, Faculty of Pharmacy, South Valley University, Qena 83523, Egypt

## Abstract

There is a need to synthesize eco-friendly nanoparticles with more effective and potent antibacterial activities. A green and cost-effective method for the synthesis of silver nanoparticles (AgNPs) using *Thymus vulgaris*, *Mentha piperita*, and *Zingiber officinale* extracts was developed. The analytical instrumentation, namely, UV/Vis, absorption spectroscopy, FTIR, and scanning electron microscopy (SEM), was used to determine the developed AgNPs, confirming the functional groups involved in their reduction. Acidic molybdate, DPPH, and FRAP regents were reacted with AgNPs extract to evaluate their antioxidant, scavenging, and oxidative activities. The agar well diffusion method was used to determine the antibacterial potential of AgNPs extracts using clinical isolates. The developed AgNPs showed peaks at 25 cum\Diff, 50 cum\Diff, and 75 cum\Diff, respectively, of 16.59 ± 0.78, 45.94 ± 1.07, and 81.04 ± 0.98 nm, for *Thymus vulgaris*, *Mentha piperita*, and *Zingiber officinale*. SEM revealed uniform prepared and encapsulated AgNPs by plant extracts matrix. The FTIR shows the involvement of amide (-CO-NH_2_), carbonyl (-CO), and hydroxyl (-OH), which resulted in the reduction of AgNPs. The AgNPs extract showed significantly higher TAA, DPPH, and FRAP values than free AgNPs and plant extract (*p* < 0.05). Antibacterial of AgNPs extracts revealed various degrees of inhibition zones against *Escherichia coli*, *Acinetobacter baumannii*, and *Staphylococcus aureus*. The developed AgNPs extract showed acceptable antioxidant activities and noticeable antibacterial potential. The prepared green synthesized AgNPs showed a promising antibacterial activity against four multidrug-resistant clinical isolates, *Escherichia coli*, *Acinetobacter baumannii*, and *Staphylococcus aureus*. Further, fractionated extracts other than crude extracts will be utilized in the preparation of AgNPs to get more efficient antibacterial activities for future work.

## 1. Introduction

Recently, nanotechnology has become one of the most important technologies from the last centuries up to date [1, 2]. The nanoparticle's delivered therapies have specific targeting, accompanied by diminishing the required high doses and subsequently fewer expected drawbacks of a drug [2, 3]. Related to the efficacy of a therapy, nanoparticles of drug delivery can improve a drug's bioavailability at specific targets in the body, as well [4]. Generally, two techniques are used as methods for the preparation and synthesis of the required nanoparticles, namely, top-down and bottom-up synthesis methods [5]. In the top-down method, a destructive method, the bulk materials, or larger molecules, are first decomposed into smaller ones and then transformed into the target size of nanoparticles. Physical vapor deposition, thermal decomposition, mechanical methods, ball-milling, lithographic, laser ablation, and sputtering methods have all been used [5, 6]. On the other hand, in the bottom-up method as a constructive method, the nanoparticles are formed from relatively smaller substances after being treated with chemical vapor deposition, sol-gel, spinning, pyrolysis, and/or biological synthesis.

In the field of biological applications of the bottom-up technique, the procedures involve the synthesis of nanoparticles incorporated from plant extracts. This is a branch of phytonanotechnology, which has been shown as a new field for the synthesis and utilization of nanoparticles as an eco-friendly, simple, and cost-effective technology. Different techniques have been applied to extract certain antioxidants from their plant parts, such as shaking with a suitable extractor solvent, homogenization at high speed, and ultrasound-, maceration-, stirring-, and microwave-assisted extraction [7–10]. Some of these plant extracts containing antioxidant compounds are greatly affected by the extraction technique used in their preparation. Therefore, the selection of certain extraction strategies depends on the simplicity of the extraction technique and its convenience for the proposed study [7]. Recently, other techniques and methods have been developed, characterized, and optimized to synthesize different types of nanoparticles. Basically, they fall into three categories: physical, chemical, and biological methods [3]. The hazards associated with the chemicals that accompanied the physical and chemical methods, as well as the fact that they may be more expensive and less available in comparison to the biological method, were responsible for limiting their use. Utilizing metallic nanoparticles is commonly used in nanomedicine for drug delivery and in the treatment of many physiological troubles and tumors. Amongst them, silver salt nanoparticles, AgNPs, are less toxic to mammalian cells than other metal nanoparticles. Due to their relatively small size, they can easily penetrate the cell through the cell membrane and serve as a potential antimicrobial agent [11, 12]. In terms of the reducing agents used, AgNPs can be synthesized using biological methods for maximum safety and efficacy [12–14].

With a safe and convenient biological synthesis of green AgNPs, phytonanotechnology has the advantage of safe synthesis and applications. On the other hand, the applications of nanotechnology accompanied by natural antioxidant plant extracts could be utilized in the management of different diseases such as tumors, atherosclerosis, arthritis, diabetes, and other ageing diseases [7, 15–17]. Through the proposed project, aqueous extracts of three common medical plants, namely, *Thymus vulgaris* (Lamiaceae), *Mentha piperita* (Lamiaceae), and *Zingiber officinale* (Zingiberaceae), were used as a green reducing agent to synthesize AgNPs having antibacterial activities against four clinical antibiotic-resistant bacterial isolates, one Gram-positive, namely, *Staphylococcus aureus* (abscess), and three Gram-negative, namely, *Proteus mirabilis* (abscess), *Escherichia coli* (urine), and *Acinetobacter baumannii* (sputum).

In the previous works we cited in this paper, some researchers tried to synthesize and characterize silver nanoparticles (AgNPs) from individual plant extracts, namely, *Zingiber officinale*, *Mentha pulegium*, *Thymus kotschyanus*, or *Thymus vulgari*s [18–22]. In some of them, they used a large amount of the extracts [19, 22] or sophisticated method for the preparation of the medicated NPs [18, 21]. All of these papers did not mention a test for antioxidants activities of the prepared medicated NPs, but they explained only the expected antimicrobial and antifungal behaviors of the loaded plant extracts test; moreover, they limited their applications in the lab. In our paper, we aimed to introduce a new and applicable study with many lab tests and real clinical applications on some known and confirmed drug-resistance strains to prove the validity of the study to be antibacterial containing medication NPs.

## 2. Materials and Methods

### 2.1. Plant Extracts

The selected plants' leaves of *Thymus vulgaris* (Lamiaceae) and *Mentha piperita* (Lamiaceae) and the roots of *Zingiber officinale* (Zingiberaceae) were purchased from the local markets of Saudi Arabia.

### 2.2. Chemicals

Silver nitrate, AgNO_3_, was obtained from VBBN Company (Hong Kong, China, 99.9% purity) and was used without purification. The stock solution of 1.0 M was prepared by adding 16.9 mg of AgNO_3_ crystals into one liter of distilled water. Ammonium molybdate reagent and hydrated ferric chloride (FeCl_3_·6H_2_O) were obtained from Merck (Darmstadt, Germany). The following chemicals were purchased: 2,2-diphenyl-1-picrylhydrazyl reagent (DPPH), 2,4,6-tris(2-pyridyl)-s-triazine (TPTZ), gallic acid, and Trolox (Sigma, USA). Standard control pure powders of chloramphenicol (2.5 mg/mL) and clotrimazole (5 mg/mL) were purchased from Sigma-Aldrich (USA). The remainder of the chemicals, solvents, and reagents utilized in this investigation were of analytical quality and were obtained from local Saudi companies.

### 2.3. The Clinical Microorganisms

Bacterial clinical isolates were generously provided by Buraidah Central Hospital in Qassim Province, Saudi Arabia. The samples were anonymously brought in Petri dishes (with no information about the patients); *Proteus mirabilis* (abscess), *Escherichia coli* (urine), *Acinetobacter baumannii* (sputum), and *Staphylococcus aureus* (abscess) were the antibiotic-resistant bacteria provided. Microbial cultures were reidentified again microscopically and microbiologically in our labs using routine tests [23]. The working bacterial specimen was adjusted to the McFarland standard (about 10^8^ CFU/mL) before the experiment.

### 2.4. Instrumentation

The bioreduction of AgNO_3_ in aqueous solution was monitored by assaying the full spectrum of the solution within the range of 380–680 nm using ultraviolet-visible (UV-Vis) spectrophotometry (Jasco, UV-630, Japan), supported by two matched quartz cuvettes. The color change in the reaction mixture (Ag^+^ in a solution and plant extracts) was recorded through visual observation. The other characterization of the formed AgNPs was done at room temperature by using a Shimadzu particle size analyzer (company, city, and country), a surface characteristic using a scanning electron microscope (JEM-1230, Joel, Japan), and a Fourier transform infrared (FTIR) spectroscopy (company, city, and country) used to study to investigate the chemical functional groups of formed AgNPs involved in the bioreduction of silver nanoparticles in the region of 4000–500 cm^−1^ at a resolution of 1.0 cm^−1^.

### 2.5. Preparation of Aqueous Extracts

The dried leaves of *Thymus vulgaris* and *Mentha piperita* and the roots of *Zingiber officinale* were cleaned carefully, washed with distilled water, and then dried in the dark for three days; they were sliced and cut into fine pieces. The dried fine species were ground carefully into fine powder. One gram of each fine powder was added to 100 mL of a beaker containing distilled water and stirred at 30°C for 2 days. These aqueous extracts were filtered twice, initially through the Whatman 41 filter paper and finally through a 0.22 *μ*m syringe filter, just before they were incorporated into the AgNO_3_ solution. When the final sterilized extracts were ready, they were kept in the refrigerator at 4°C wrapped in aluminum foil so they could be used again.

### 2.6. Synthesis of AgNPs Incorporated with Extracts

Trial experiments were conducted to adjust the final preparations. In brief, the aqueous extract of *Thymus vulgaris* and *Mentha piperita*, 5 mL, was taken along with 95 mL of distilled water and 16.9 mg (100 *μ*L) of AgNO_3_. The mixture was kept for 24 hours on the magnetic stirrer to obtain the AgNPs. In contrast, the aqueous extract of *Zingiber officinale*, 50 mL, was taken along with 50 mL of distilled water and the same amount of AgNO_3_. The molar concentration was calculated depending on the amount of Ag ion in the solution. A control of AgNPs using sodium citrate was prepared similarly.

### 2.7. Spectrophotometric Characterization of AgNPs

The color change of *Thymus vulgaris*, *Mentha piperita*, and *Zingiber officinale* extracts and the reduced AgNPs were detected visually. The bioreduction of Ag^+^ in an aqueous solution was monitored by measuring the spectrum of the solution within the range of 380–680 nm using a UV-Vis spectrophotometer and a quartz cuvette with distilled water as the reference. To study the stability of colloidal AgNPs solution, the solution was kept at room temperature for one week. During this period, the UV-Vis spectrum of the solution was measured at different intralaboratory intervals, after half an hour, 24 hours, and one week. The color and pH of the solution were also checked at regular intervals, which hardly showed any changes.

### 2.8. Particle Size Determination

The selected plant extracts were used to reduce AgNPs and were analyzed to determine their diameter using a Shimadzu particle size analyzer at 25°C. The resulting nanoparticles were subjected to a laser beam using a laser particle size analyzer (SLAD-400 from Shimadzu, Japan) with a wavelength of 623 nm and an angle of 90°.

### 2.9. Morphology and Characterizations of AgNPs

A scanning electron microscope (SEM) (Jeol, Akishima, Tokyo, Japan) was used to investigate all types of AgNPs morphology and particle size. In a carbon-coated copper grid, drops of freshly prepared AgNPs-EX solution were spotted and left to dry at room temperature. A 10–100 K magnification microscope power and an accelerating voltage of 100 kV were used to display the morphology of the samples. The Fourier transform infrared spectroscopy (FT-IR) (Nicolet™ iS50 FTIR Spectrometer, Thermo Scientific Co., Twin, USA) measurements were used to analyze the compatibility of the biomolecules associated with AgNPs formation. It was measured with a Bruker Tensor 27 FTIR spectrophotometer in the wavelength range of 4000–400 cm^−1^ [24].

### 2.10. Screening of Antioxidant Activity

As a comparable assay, three *in vitro* methods were conducted to evaluate the total antioxidant activities of the selected extracts incorporating AgNPs. The methods were conducted in triplicate experiments, and the antioxidant activity was calculated using standard calibration curves of known standards for each method.

#### 2.10.1. Total Antioxidant Capacity (TAC)

The TAC of the extracts and the prepared AgNPs was conducted according to the reported methods [25–27], with slight modifications. Briefly, the freeze-derided extracts were diluted with distilled water to prepare different working solutions (200 *μ*g/mL). Then, 200 *μ*L of each of these samples' solutions was transferred separately into 10 mL capped plastic tubes and mixed vigorously with freshly prepared acidic solutions of ammonium molybdate reagent (0.6 M sulfuric acid, 28 mM sodium phosphate, and 4.0 mM ammonium molybdate, 2 mL). The contents of the tubes were incubated in a water bath at 85°C for 95 min and cooled down to room temperature. Then the absorbance of the arising blue color was measured spectrophotometrically at 695 nm against blank experiments, which were treated similarly. Ascorbic acid (5.0–100.0 *μ*g/mL and 0.0284–0.568 mM, resp.) was used as the standard. The TAA is expressed as equivalent to the ascorbic acid calibration curve. By measuring the absorbance at 695 nm (*A*_695_) and assessing it intraday using five replicates, the molar absorptivity coefficient of the calibration curve, the concentration range for linearity and validity of Beer's law, and the precision of the technique were determined.

#### 2.10.2. DPPH Radical Scavenging Activity

Extract solutions with 50–200 g/mL were mixed with a solution of fresh-made DPPH (0.3 mg/L) in separate 10 mL plastic tubes. Then, the mixture was put into a 10 mL capped plastic tube. The test tube was then incubated in the dark for 30 min at room temperature to develop the violet color. The decrease in absorbance was measured at 515 nm using the spectrophotometer. The percentage inhibition of radicals was calculated using the following formula:

(1) $$\begin{array}{c} \%\text{inhibition}=\frac{A_{\text{control}}-A_{\text{sample}}}{A_{\text{control}}}\times 100\%, \end{array}$$

where *A*_control_ is the absorbance, UA, of the DPPH reagent solution without the extract and *A*_sample_ is the absorbance of the sample with DPPH solution; the half-maximal inhibitory concentration (IC_50_) was reported as the amount of antioxidant required to decrease the initial DPPH concentration by 50%. All tests were performed in triplicate, and the analytical figure of the merit was plotted using the average of three determinations ± standard deviation.

#### 2.10.3. Ferric Reducing Antioxidant Power (FRAP)

The antioxidant capacity of samples was determined according to the Benzie and Strain method [28] with slight modifications [29, 30]. The method is based on the reduction of the Fe^3+^-TPTZ complex to the ferrous form at low pH. The fresh working solution (FRAP reagent) was prepared by mixing 25 mL of acetate buffer (300 mM, pH 3.6), 2.5 mL of TPTZ solution (10 mM in HCl (40 mM)), and 2.5 mL of FeCl_3_·6H_2_O solution (20 mM) and then warmed at 37°C before being used. Aliquots of 0.1 mL of the diluted working solution of AgNPs extract samples (50–200 *μ*g/mL) were added to 4.0 mL of FRAP reagent and mixed well to form a mixture. The mixtures were incubated at 37°C for 10 min in the dark, and then the absorbance was measured at 593 nm against the blank experiments, which were treated similarly using distilled water instead of the AgNPs extracts. The results, obtained from triplicate analyses, were expressed as an aqueous solution of ferrous sulfate (FeSO_4_·7H_2_O) and derived from a calibration curve of the standards (20∼1,000 *μ*M).

### 2.11. Antibiotics Sensitivity Profile

Clinical isolates were subjected to antibiotic sensitivity tests, following the Clinical and Laboratory Standards Institute's guidelines [31]. The test was carried out using the MicroScan WalkAway system (96 plus, Bekman Coulter, USA) to evaluate the possible resistance or susceptibility of the isolated clinical strains. A referenced broth microdilution assay was employed. An overnight culture broth of bacterial isolates was adjusted to be comparable to 0.5 McFarland, the adjusted bacterial suspension was injected into the panel, and the necessary antibiotics were also loaded. The panel was placed in the MicroScan WalkAway system for 24 hours of incubation, and the results were recorded by the system itself.

### 2.12. Evaluation of Antibacterial Activity

The expected antibacterial activity of biogenic AgNPs extract was investigated using clinical bacterial isolates in a standard agar well diffusion assay [32]. A standard antibiotic (chloramphenicol, 2.5 mg/mL) was used as a positive control. As controls, standard AgNO_3_ and AgNPs solutions prepared with sodium citrate were also used. Before the antibacterial screening, the previously prepared bacteria were subcultured and adjusted. A group of 25 mL glass bottles containing autoclaved nutrient agar were loaded into presterilized Petri dishes and left to solidify at ambient temperature. On the surface of the plates, a sterile cork borer (6 mm) was used to drill four wells. The agar plates were spread with 100 *μ*L of a standardized culture of the organisms (adjusted to 0.5 McFarland, 10^7^ CFU/mL). Then 50 *μ*L of AgNPs extract and control was loaded into the wells, 50 *μ*L of chloramphenicol (2.5 mg/mL) was loaded into the other wells, and they were incubated overnight at 35°C. The inhibition zone was measured after incubation. The diameter of the inhibition zone (mm) was used to represent antibacterial activities, and the test was repeated three times to obtain the mean reading (mm ± SD). The antimicrobial inhibitory effect of controlled AgNPs was previously investigated and adjusted to the minimum inhibitory effects at a concentration of 1.0 mM (during the preexperimental phase). If the zone was less than 10 mm, it was considered weak antibacterial activity, moderate if it was between 10 and 13 mm, and high if it was 13 mm or more.

### 2.13. Determination of the Minimal Inhibitory Concentration (MIC)

The clinical isolates that showed noticeable susceptibility to the tested compounds with the agar diffusion assay were tested using the minimal inhibitory concentration (MIC) following the microdilution method [33]. Serial twofold dilutions were made for all the three tested compounds (the extracts alone and the extracts with AgNPs and AgNPs alone), in descending concentrations of 2.26, 1.13, 0.56, 0.28, 0.14, 0.0716, and 0.04 mg/mL, respectively. Compounds were diluted with dimethyl sulfoxide (DMSO) 99%.

DMSO showed in the preexperimental phase no effect on microbial growth. Therefore, serial twofold dilutions were also prepared for DMSO and served as negative controls. An antibiotic (chloramphenicol 2.5 mg/ml) was serially diluted to serve as a positive control. Then, 100 *μ*L of sterile nutrient broth was added to all the wells. Plates were then incubated overnight at 37°C. Bacterial growth was determined by absorbance at 959 nm using the iMark Absorbance Microplate Reader (BIO-RAD Inc.). The whole test was repeated thrice to get the accurate MIC.

### 2.14. Determination of Minimal Bactericidal Concentration (MBC)

MBC was evaluated by subculturing 50 *μ*L of the test dilution from each of the 96 wells on nutrient agar plates. MBC was defined as the maximum dilution that revealed no single bacterial colony. MBC/MIC was determined [32].

### 2.15. Statistical Analysis

The results in this study are presented as mean ± standard deviation (SD). The one-way ANOVA (analysis of variance) test was performed to determine if there were any statistically significant differences between the microorganisms tested, and SPSS 14.0 (SPSS Inc., Chicago, USA) was used to perform the data analysis.

## 3. Results and Discussion

AgNPs have attracted attention for their health benefits, environment greenness manners are still required [32]. The initial studies demonstrated their effects as antimicrobials due to the sliver (Ag^+^) that will be released from the prepared AgNPs surface [34]. The Ag^+^ release rate is a function of the nanoparticle size, as the smaller particles have a faster release rate, as well as a higher temperature, which increases the dissolution and their bioavailability. In the reported literature [35–38], different suggested mechanisms have been introduced for the synthesis of AgNPs via natural plant extracts. Releasing or sharing of electrons from plant extract is responsible for the reduction of ionized positive ions, Ag^+^, and the phenolic compounds that contain OH, C=O, and CH groups are considered the electron enrichment suppliers. Another factor was mentioned and had a vital role in the increased rate of prepared AgNPs, which is the elevated temperature or radiated from the surrounding environmental light. It was suggested that the mechanism of their antibacterial activity is the binding of negative charge in the cell wall of the bacteria, with positive charge ions of silver, which could lead to the death of the bacterial cell [18, 39]. Consequently, in the present work, the aqueous extracts of *Thymus vulgaris*, *Mentha piperita*, and *Zingiber officinale* were considered the reducing agents that reduced AgNO_3_ to AgNPs.

### 3.1. Synthesis of AgNPs Incorporating Plant Extracts

**Figure 1:**
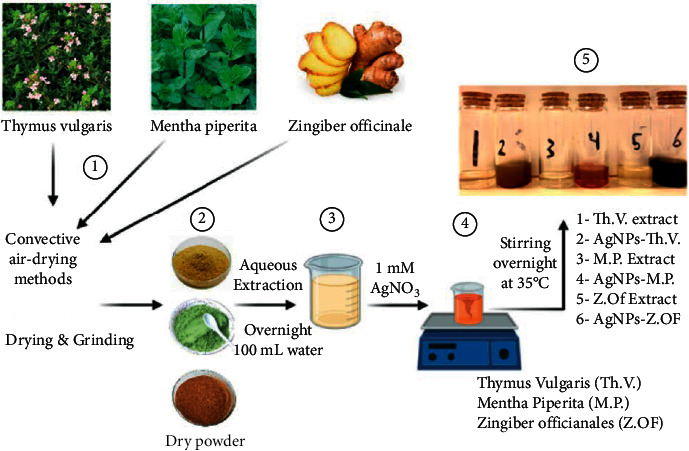
Schematic diagram for the aqueous extraction of the selected plant and AgNPs reduced with the extracts.

As previously stated [19], the influence of reducing agents and their stability on the physicochemical characteristics of AgNPs was deemed to be relevant criteria in this work. As a result, the reaction conditions were originally optimized using a variety of different kinds and amounts of plant extracts, as well as various concentrations of silver nitrate, in order to get stable nanoparticles. The colors of the original materials altered reproducibly at the endpoint of the final synthesized AgNPs during the green synthesis of the natural extracts reduced AgNPs. The gray or white initial hues of *Thymus vulgaris*, *Mentha piperita*, and *Zingiber officinale* aqueous extracts have been altered to olive green, dark olive green, and dark orange, respectively, indicating the creation of decreased AgNPs, as illustrated in Figure 1. These results were also agreed with our previous results, which showed changing color from yellow to red color when reducing *Allium cepa* L. to AgNPs reduced with *A*. *cepa* L. [40]. Moreover, other confirmations when reducing the AgNO_3_ with *Salsola vermiculata* also showed green and stabilized AgNPs red color AgNPs after reduction with Salsola [41].

In the previous methods, different types of tedious and expensive materials for preparing AgNPs, as well as the concentration of the selected extracts, were used, from the side of AgNO_3_ and/or extracted solutions [42]. Moreover, in the present study, we used a simple and rapid method for the synthesis of plant extracts incorporated with AgNPs by using a simple and readily available tool such as a magnetic stirrer.

### 3.2. Characterization of AgNPs

Different analytical and spectroscopic techniques are used to characterize the morphology, shape, size, and stability or aggregation of the formed monodisperse AgNPs.

### 3.3. Spectrophotometric Characterization

UV-Vis spectrophotometry is the principal tool for elucidating the production of AgNPs during the early synthesis phase [43]. This test demonstrated that the smaller (spherical) AgNPs absorbed the light photons at a wavelength of 400 nm, while the bigger AgNPs had a redshift and more wide peaks. These findings demonstrated the stability of manufactured AgNPs containing natural extract, as the peaks began to weaken and widen with the formation of secondary peaks at longer wavelengths as the particles aggregate. Additionally, the color change in the solutions of *Thymus vulgaris*, *Mentha piperita*, and *Zingiber officinale* extracts during synthesis is size and shape-specific, confirming the progress of their reduction to AgNO_3_ throughout synthesis processes. The quantity of aqueous extracts utilized and their wavelengths, in nm, are listed in Table 1. These results were confirmed by Sadeghi et al. [44], who showed that the colorless AgNO_3_ solution was turned from yellow to brown to deep red as an indication of the formation of AgNPs. Moreover, the appearance of the brown color is due to the excitation of the surface plasmon resonance (SPR). Further, the SPR absorbance was particularly sensitive to the type, size, and shape of the particles formed and their interparticle.

### 3.4. Particle Size Determination

**Figure 2:**
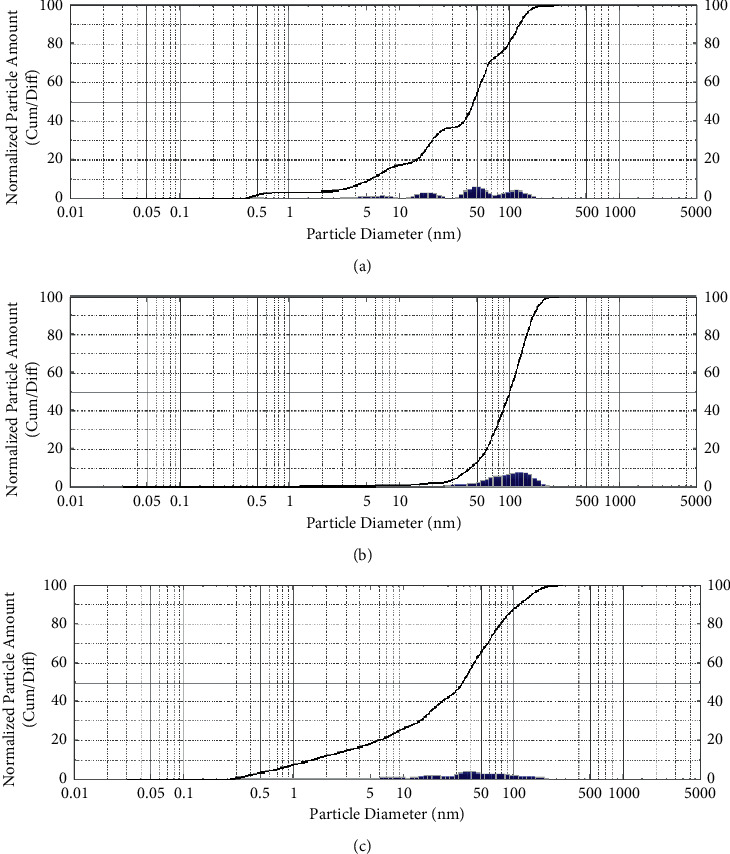
The graph shows the particle diameter of AgNPs reduced by *Thymus vulgaris* (a), *Mentha piperita* (b), and *Zingiber officinalis* (c).

Using the Shimadzu particle size analyzer, Figures 2(a)–2(c) depict the patterns of decreased AgNPs at the peaks of 16.59 ± 0.78, 45.94 ± 1.07, and 81.04 ± 0.98 nm, respectively, for *Thymus vulgaris*, *Mentha piperita*, and *Zingiber officinale*. A substantial change in size was detected when the size of spherical AgNPs from the same sample with various natural extracts was examined using a particle size analyzer. These differences were within the range of AgNPs nanosized, reported to be between 40 and 145 nm [45].

For *Thymus vulgaris*, Figure 2(a) shows the pattern of AgNPs with the peak at 25 cum\Diff, 50 cum\Diff, and 75 cum\Diff of 16.595, 45.936, and 81.045 nm, respectively. The median diameter was 45.936 nm, while the mean volume was 31.423, and the standard deviation was 0.568 nm. For *Mentha piperita*, Figure 2(b) shows the peak at 25 cum\Diff, 50 cum\Diff, and 75 cum\Diff of 67.084 nm, 99.224 *μ*m, and 134.219 nm, respectively. The median diameter was 99.224 nm, while the mean volume was 87.597, and the standard deviation was 0.291. For *Zingiber officinale*, Figure 2(c) shows the peak at 25 cum\Diff, 50 cum\Diff, and 75 cum\Diff of 8.782, 33.470, and 65.602 nm, respectively. The median diameter was 33.470 nm, while the mean volume was 20.342, and the standard deviation was 0.703 nm.

### 3.5. Particle Size Morphology and Characterization

**Figure 3:**
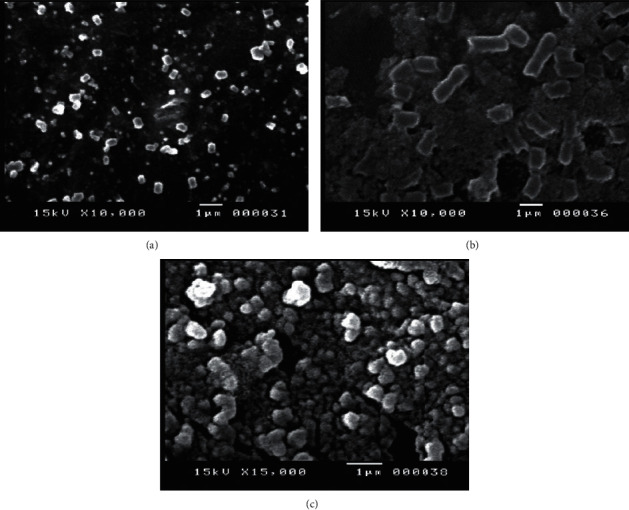
SEM for the prepared AgNPs incorporating natural aqueous extract of (a‐c) *Thymus vulgaris, Mentha piperita, and Zingiber officinalis.*

The scanning electron microscopy (SEM) was employed in this investigation to describe the surface morphology, size, aggregation, and dispersion of nanoparticles using electron beams as imaging probes. It generates high-resolution pictures at the nanoscale scale, demonstrating the biomatrix's function in the encapsulation of AgNPs, and is used to determine the size of nanoparticles [46, 47]. The SEM for the prepared AgNPs incorporating natural aqueous extract revealed the encapsulation of AgNPs by the plant extracts matrix. This clearly indicates the spherical morphology of AgNPs coated by the extract (Figure 3). SEM images showed round to cubic shape, particle with rode-like shape, and cubic structure for AgNPs reduced with *Thymus vulgaris*, (Figure 3(a)) *Mentha piperita*, (Figure 3(b)) *and Zingiber officinale* (Figure 3(c)). Moreover, the SEM confirmed the formation of the green synthesized and stabilized AgNPs. The formulated AgNPs were clearly in the form of separated NPs; also, no aggregations were observed through the imaged AgNPs. The SEM recorded size differences in the prepared AgNPs compared with that obtained by the size analyzer. These sizes also are suitable for the interaction and internalization of cells. The particle diameters measured by the size analyzer are smaller than those measured by SEM imaging, which is thought to be related to the presence of the coating layer around AgNPs, which also reduces overall particle density. The size analyzer calculates average particle sizes, which differ dramatically from those obtained by SEM. Because the nanosuspension may agglomerate and impact the average distribution of size, the average size estimations of AgNPs may fluctuate and not be reproducible [24, 48].

Fourier transform infrared spectroscopy (FTIR) was used to characterize the role of natural extracts in their degradants or other coeluted components involved in reducing or capping of AgNPs and/or present on the surface of AgNPs. Moreover, the study revealed the potential biomolecules that participated in the bioreduction of silver and stabilization of AgNPs. The interaction of the nondestructive IR radiation with the bonding in the formed molecules takes the form of stretching and bending vibrations (in the region of 4000–400 cm^−1^) [12]. The FTIR spectral analysis shows the involvement of amide (-CO-NH_2_), carbonyl (-CO), and hydroxyl (-OH) functional groups responsible for the reduction, capping, and stability of AgNPs [49, 50]. The reduction and formation of AgNPs were investigated (FTIR).

**Figure 4:**
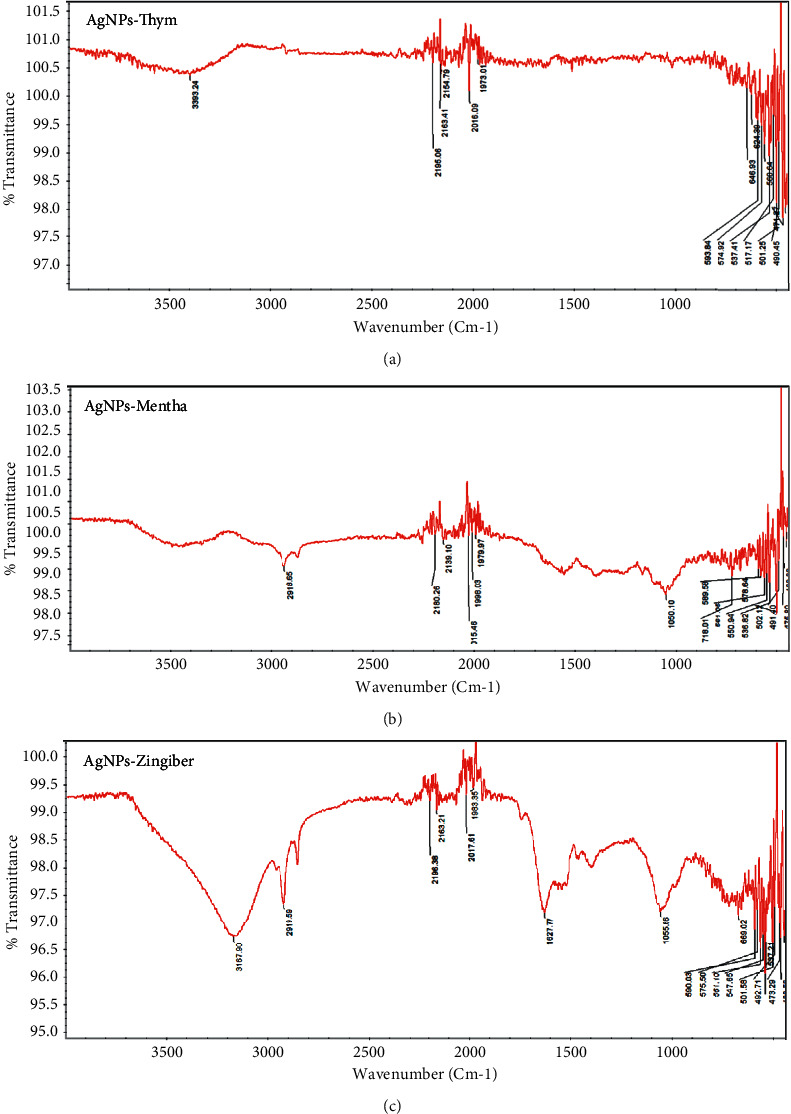
FTIR spectroscopy of AgNPs incorporating natural aqueous extract of *Thymus vulgaris* (a), *Mentha piperita* (b), and *Zingiber officinalis* (c).

Figure 4(a) shows the bands of *Thymus vulgaris* extract and its AgNPs. The band at 3393.24 cm^−1^ indicates the presence of an OH bond. The peak at 2195.05 cm^−1^ indicates the presence of CH stretching vibration related to CH_2_ and CH_3_ groups. The peak at 1973.01 cm^−1^ is due to C=O stretching. Therefore, the reduction in peak intensity could indicate the formation of AgNPs that its peak has occurred at 646.93 cm^−1^. The weak peaks at 595.84 cm^−1^ and 574.92 cm^−1^ are interrelated to the OH bond of the phenolic group. As shown in Figure 4(b), the FTIR spectrum of *Mentha piperita* aqueous extract incorporated into AgNPs indicates the presence of the AgNPs on the biomolecules because the band absence at 2400 cm^−1^ from *Mentha piperita* extract (attributed to the C=O group) in the FTIR spectrum of *Mentha piperita* extract incorporated into AgNPs confirms the involvement of C=O groups in the decoration step of *Mentha piperita* extract with AgNPs.  Figure 4(c) shows the FTIR analysis of *Zingiber officinalis* extract and AgNPs synthesized via ginger extract. The spectrum at 3167.90 cm^−1^ belongs to the OH stretch bonds. The weak peaks at 2919.59 cm^−1^ are related to the OH stretching of the carboxylic acid group [51]. The bands at 1627.77 cm^−1^ are related to the presence of the C=O stretch of alkyne, and the band at 1610 cm^−1^ is related to the C=O stretch [52]. The band appears at 1055.66 cm^−1^ with CH_3_ symmetric bonds in the alkene group. Other weak peaks after this band are related to stretching C=O, and OH phenolic bonds have been seen at 617 cm^−1^ and 618 cm^−1^ [22].

We demonstrated that the extracts are responsible for the decrease of the produced AgNPs based on the FTIR findings of the extracts and the AgNPs created through extracts. The change in the bond shows that extracts offer the necessary electrons for the production of AgNPs through OH bond splitting. Additionally, this bond breakdown results in the attachment of extracts to AgNPs. As a result, the FTIR indicates that the extracts are adsorbed to the surface of AgNPs and operate as a capping agent in addition to the presence of coeluted components in the extract.

### 3.6. Antioxidant Activity Screening

The total antioxidant capacity (TAC) of the AgNPs extract reducing species was evaluated based on the molar absorptivity coefficient of the formed phosphomolybdenum complex. An appropriate reducing power of standard ascorbic acid was used to make calibration curves that were used to compare the standard ascorbic acid with the samples. The determined molar absorptivity coefficient and the concentration range for the validity of Beer's law of the phosphomolybdenum method applied to the determination of ascorbic acid, respectively, were (3.4 ± 0.2) × 10^3^ M^−1^Cm^−1^ and 0.0284–0.568 mM, with a correlation coefficient (*R*^2^) of 0.998. The difference between the free aqueous extract compared with the incorporated AgNPs showed a significantly higher TAA in the case of extracts-AgNPs than the free AgNPs and plant extract (*p* < 0.05).

**Figure 5:**
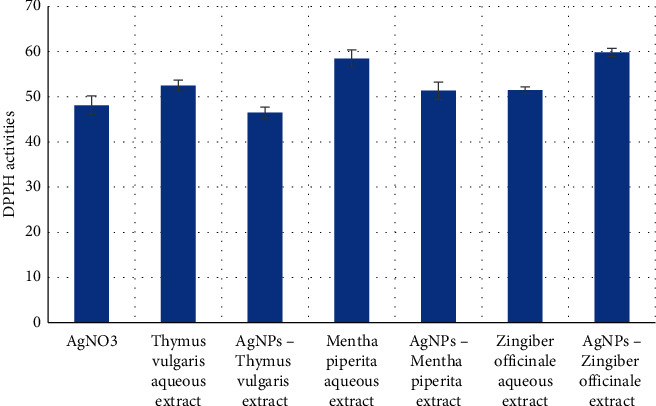
DPPH radical scavenging activities of the tested compounds.

Due to its stability as an organic free radical, DPPH has radical scavenging action. The scavenging activity of the produced AgNPs was determined in this work by measuring the reduction in the absorption peak of its purple hue at 517 nm after an electron of hydrogen radical from a reducing species discolored it to yellow [53, 54]. The DPPH scavenging capabilities of the free extracts and AgNPs extracts at various concentrations are shown in Figure 5. At a probability of *p* < 0.05, it is demonstrated that there are significant variations in the values of the tested items.

The results obtained thus indicate that AgNPs extract has antioxidant activity, indicated by their scavenging abilities evaluated against the DPPH free radical. The selected plant extracts have been reported to contain flavonoid and isoflavonoid glycosides, which are well-known antioxidants.

**Figure 6:**
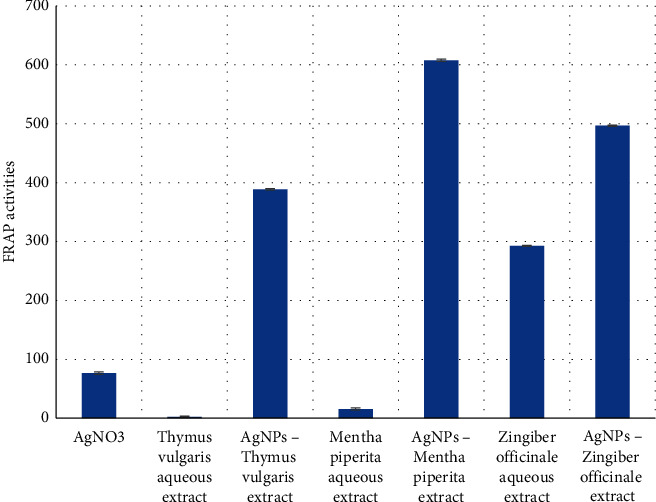
Ferric reducing antioxidant power (FRAP) of the tested compounds.

The Ferric reducing antioxidant power (FRAP) assay is simple and reliable in that it measures the reducing power of an antioxidant reacting at low pH with the Fe(III)-TPTZ complex and can be monitored at 593 nm after producing the colored Fe(II)-TPTZ complex. A higher absorbance power indicates a higher ferric reducing power. In the present work, the FRAP values for free extracts, AgNO_3_, and AgNPs extract were estimated, as shown in Figure 6.

All types of AgNPs showed singnificantly (*p* < 0.05) antioxidant activities higher than either the AgNO3 or free plant extracts, indicating, indicating that incorporation of free extract with AgNPs possesses a higher reducing capacity than their free form due to the synergistic activities [12, 13, 20].

### 3.7. In Vitro Antimicrobial Activity of Green Synthesized AgNPs

The clinical isolates' antibiotic sensitivity test results are provided in (Table 2), indicating their probable resistance to several common antibiotics. Only *Staphylococcus aureus* was shown to be resistant to two antibiotics, whereas the other three bacteria are multidrug-resistant. The multidrug-resistant pathogens are a serious concern in a large number of long-term care facilities and hospitals worldwide [55]. Phytonanotechnology, on the other hand, is gaining a lot of attention because of its quick, eco-friendly, nontoxic, and cost-effective process [56].

The antibacterial activity of AgNPs extracts is summarized in Tables 3 and 4. The agar well diffusion test revealed a substantial advantage for *Thymus vulgaris* AgNPs over *Thymus vulgaris* solution (1 M) against *Staphylococcus aureus* (*p* < 0.05). Additionally, *Mentha piperita* AgNPs showed a strong antibacterial activity to varying degrees against all pathogens examined except *Proteus mirabilis*. Furthermore, AgNPs from *Zingiber officinale* showed substantial action against *Staphylococcus aureus*, *Escherichia coli*, and *Klebsiella pneumonia*, respectively. These findings were achieved in comparison to extract solutions alone. The MIC and MBC values indicated the effectiveness of AgNPs extracts since they were much lower than those of non-AgNPs extracts, except for *M*. *piperita*, which showed some similarity. The MIC and MBC values supported the effectiveness of AgNPs extracts (*Thymus vulgaris* AgNPs, *Mentha piperita* AgNPs, and *Zingiber officinale* AgNPs) since they were much lower than those of non-AgNPs extracts, except for *Mentha* piperita. The MBC/MIC values for AgNPs extracts were 2 and 4, respectively, while those for non-AgNPs extracts varied from 2 to 16. It is known that when the MBC/MIC ratio is less than 4, the extract is deemed bactericidal; when the MBC/MIC ratio is more than 4, the extract is regarded as bacteriostatic [57]. Thus, their bactericidal effects were enhanced when AgNPs were combined with plant extracts.

The present research sought to determine the antibacterial activity of AgNPs extracts and non-AgNPs extracts at low concentrations (about 1 m molar). Previous investigations employed larger concentrations, and their findings on *Thymus vulgaris* AgNPs, *Mentha piperita* AgNPs, and *Zingiber officinale* AgNPs are generally consistent with ours. *Thymus vulgaris*-biosynthesized AgNPs showed exceptional antibacterial efficacy against *Escherichia coli* and *Staphylococcus aureus* [58]. AgNPs from *Mentha piperita* have been demonstrated to exhibit antibacterial activity against *Bacillus cereus*, *Staphylococcus aureus*, *Escherichia coli*, and *Salmonella typhimurium* [59]. Additionally, antibacterial action against *Staphylococcus aureus* and *Escherichia coli* was increased by *Zingiber officinale* AgNPs [60]. AgNPs have antibacterial action in general; however, the mechanism by which they do so is uncertain. However, the mechanism by which they are antibacterial has been postulated to be the binding of negative charge on the bacteria's cell wall to positive charge silver ions, which may result in the bacteria's cell death [18].

The antibacterial activity of AgNPs-sodium citrate against a broad spectrum of microorganisms has been well known for decades. On the other hand, medicinal plants have a well-documented antibacterial capability [61]. The extract and devices based on AgNPs have found widespread use in water, air modification, food production, preservatives, cosmetics, clothing industry, and biomedicine [19, 62, 63]. Several mechanisms have been reported for the antibacterial activity of AgNPs, including direct damage to the bacterial cell membrane. The release of Ag^+^ ions causes the generation of reactive oxygen species, which leads to increased membrane permeability and DNA damage [64]. It has been established that the size, shape, and surface chemistry of AgNPs significantly affect their potential for cell penetration as well as their cytotoxicity and antibacterial activity. Therefore, selecting suitable stabilizing reducing agents for efficient penetration of the nanoparticles into the bacterial cells can improve their antibacterial activity, consequently leading to a decrease in their therapeutic dose [63]. Moreover, the use of natural organic compounds, with defined and known antimicrobial activity [18–20], for the functionalization of AgNPs may potentiate their therapeutic effects, which are known as capping agents. These capping agents could also improve the colloidal stability of the AgNPs by avoiding their aggregation and significantly affect their interactions with *in vivo* components [65]. In the current study, to investigate the net hypothesis, the antibacterial activities of the biogenic AgNPs extracts were evaluated and confirmed during the present study.

The current results showed that AgNPs prepared using sodium citrate were used as a control for examination of the antibacterial effect of AgNPs solution. Prior to the further experiments, the feeble inhibitory effect was recorded at a low concentration of 1.0 mM, as well as the minimum inhibitory concentration (MIC). Then, the effect of the free selected plant extracts and their effect after being incorporated into AgNPs could be analyzed for their synergic association.

## 4. Conclusions

In the present work, we deliver AgNPs containing aqueous extracts of *Thymus vulgaris*, *Mentha piperita*, and *Zingiber officinale*, with the assistance of a simple magnetic stirrer procedure. We confirmed the purity of the natural, medicated extracts to be used as reducing agents to synthesize AgNPs, as well. The prepared medicated AgNPs were applicable as an antibacterial for some multidrug-resistant clinical isolates. For fractionating these extracts, other than crude extracts, further work will be conducted for the preparation of AgNPs to get more efficient, sensitive, and specific explanations as antibacterial and anticancer activities.

## Figures and Tables

**Table 1:** The exact amounts of AgNO_3_ and natural extracts in the synthesized AgNPs and their wavelength.

Plant	Amount of AgNO_3_, mg	Amount of aqueous extract/100 mL (mg)	Wavelength of each aqueous extract	Wavelength of synthesized AgNPs (nm)
*T*. *vulgaris*	16.9	116	394 nm	412
*M*. *piperita*	16.9	276	389 nm	433
*Z*. *officinale*	16.9	286	287	400

**Table 2:** Antibiotics susceptibility profile of clinical bacterial isolates.

Clinical isolate	Source	Standards agents resistance
*Staphylococcus aureus*	Abscess	Ampicillin, penicillin, and other beta-lactamases, tobramycin, trimethoprim/sulfa agents
*Proteus mirabilis*	Abscess	Gentamicin/ampicillin, ampicillin/sulbactam, and amoxicillin/clavulanic acid
*Escherichia coli*	Urine	Ciprofloxacin, levofloxacin, tigecycline, amikacin, ampicillin/sulbactam, and cefepime
*Acinetobacter baumannii*	Sputum	Ceftazidime, ciprofloxacin, gentamicin, levofloxacin, meropenem, tobramycin, and trimethoprim/sulfa agents

**Table 3:** Antibacterial activity of clinical strains of AgNPs extracts compared to aqueous extracts alone and standard antimicrobial drugs, shown as inhibition zone in mm ± SD at concentration 1 mg/mL.

Tested compound	Mean zone of inhibition of clinical isolates (mm)				
	*S*. *aureus*^*∗*^	*A*. *baumannii*^*∗*^	*K*. *pneumonia*^*∗*^	*E*. *coli*^*∗*^	*P*. *mirabilis*^*∗*^
*T*. *vulgaris*	—^*∗∗*^	—	—	—	—
*T*. *vulgaris* AgNPs	8.5 ± 0.7	—	—	—	—
*M*. *piperita*	—	—	—	—	—
*M*. *piperita* AgNPs	12.5 ± 0.7	8.0 ± 0.0	10.0 ± 0.0	9.0 ± 0.0	—
*Z*. *officinalis*	—	—	—	—	—
*Z*. *officinalis* AgNPs	10.0 ± 0.0	—	7.2 ± 0.3	8.2 ± 0.3	—

^*∗*^^*∗*^ *Staphylococcus aureus*, *Acinetobacter baumannii*, *Klebsiella pneumonia*, *Escherichia coli*, and *Proteus mirabilis*; ^*∗∗*^(—) = 6.0 mm well diameter (no activity), mean of three replicates ± SD.

**Table 4:** The MIC, MBC, and MBC/MIC values of *Staphylococcus aureus* clinical strain with tested compounds.

Tested compound	MIC (mg/mL)	MBC (mg/mL)	MBC/MIC
*T*. *vulgaris*	0.1412	0.2825	2.0
*T*. *vulgaris* AgNPs	0.0353	0.0706	2.0
*M*. *piperita*	0.5650	2.2600	4.0
*M*. *piperita* AgNPs	0.5650	2.2600	4.0
*Z*. *officinalis*	0.1412	2.2600	16.0
*Z*. *officinalis* AgNPs	0.1412	0.5650	4.0

## Data Availability

The data that support the results of this study are available upon request to the corresponding authors.

## References

[1] Krukemeyer M., Krenn V., Huebner F., Wagner W., Resch R. (2015). History and possible uses of nanomedicine based on nanoparticles and nanotechnological progress. *Journal of Nanomedicine and Nanotechnology*.

[2] de Jong H., Borm Paul J. A. (2008). Drug delivery and nanoparticles: applications and hazards. *International Journal of Nanomedicine*.

[3] Saba P., Maryam G., Saeid B. (2019). Green synthesis of silver nanoparticles using the plant extract of *Salvia spinosa* grown in vitro and their antibacterial activity assessment. *Journal of Nanostructure in Chemistry*.

[4] Adriano C., Shirinzadeh B., Freitas R. A., Hogg T. (2007). Nanorobot architecture for medical target identification. *Nanotechnology*.

[5] Irfan I., Ezaz G., Ammara N., Aysha B. (2020). Detail review on chemical, physical and green synthesis, classification, characterizations and applications of nanoparticles. *Green Chemistry Letters and Reviews*.

[6] Siavash I. (2011). Green synthesis of metal nanoparticles using plants. *Journal of Green Chemistry*.

[7] Musa K. H., Abdullah A., Jusoh K., Subramaniam V. (2011). Antioxidant activity of pink-flesh guava (*Psidium guajava* L.): effect of extraction techniques and solvents. *Food Analytical Methods*.

[8] Xu B. J., Chang S. K. C. (2007). A comparative study on phenolic profiles and antioxidant activities of legumes as affected by extraction solvents. *Journal of Food Science*.

[9] Contini M., Baccelloni S., Massantini R., Anelli G. (2008). Extraction of natural antioxidants from hazelnut (*Corylus avellana* L.) shell and skin wastes by long maceration at room temperature. *Food Chemistry*.

[10] Alothman M., Bhat R., Karim A. A. (2009). Antioxidant capacity and phenolic content of selected tropical fruits from Malaysia, extracted with different solvents. *Food Chemistry*.

[11] Zhao G., Stevens S. E. (1998). Multiple parameters for the comprehensive evaluation of the susceptibility of *Escherichia coli* to the silver ion. *Biometals*.

[12] Sonika D., Saurav K., Aakash G. (2021). Current research on silver nanoparticles: synthesis, characterization, and applications. *Journal of Nanomaterials*.

[13] Niknejad F., Nabili M., Daie Ghazvini R., Moazeni M. (2015). Green synthesis of silver nanoparticles: another honor for the yeast model *Saccharomyces cerevisiae*. *Current Medical Mycology*.

[14] Veeresham C. (2012). Natural products derived from plants as a source of drugs. *Journal of Advanced Pharmaceutical Technology and Research*.

[15] Temple N. J. (2000). Antioxidants and disease: more questions than answers. *Nutrition Research*.

[16] Hawar S. N., Al-Shmgani H. S., Al-Kubaisi Z. A., Sulaiman G. M., Dewir Y. H., Rikisahedew J. J. (2022). Green synthesis of silver nanoparticles from Alhagi graecorum leaf extract and evaluation of their cytotoxicity and antifungal activity. *Journal of Nanomaterials*.

[17] Jabir M. S., Saleh Y. M., Sulaiman G. M. (2021). Green synthesis of silver nanoparticles using *Annona muricata* extract as an inducer of apoptosis in cancer cells and inhibitor for NLRP3 inflammasome via enhanced autophagy. *Nanomaterials*.

[18] Shalaby T. I., Mahmoud O. A., El Batouti G. A., Ibrahim E. E. (2015). Green synthesis of silver nanoparticles: synthesis, characterization and antibacterial activity. *Journal of Nanoscience and Nanotechnology*.

[19] Kelkawi A. H. A., Kajani A. A., Bordbar A. K. (2017). Green synthesis of silver nanoparticles using *Mentha pulegium* and investigation of their antibacterial, antifungal and anticancer activity. *Journal of IET Nanobiotechnology*.

[20] Mona H., Mahdi Z. M., Abbas A., Kambiz V., Hojat V. (2018). Green synthesis of silver nanoparticles using *Thymus kotschyanus* extract and evaluation of their antioxidant, antibacterial and cytotoxic effects. *Journal of Applied Organometallic Chemistry*.

[21] Abdelhamid S. M., El-Hosseiny L. S. (2017). Combined efficacy of thymol and silver nanoparticles against *Staphylococcus aureus*. *African Journal of Microbiology Research*.

[22] Mohammadi M., Shahisaraee S. A., Tavajjohi A. (2019). Green synthesis of silver nanoparticles using *Zingiber officinale* and *Thymus vulgaris* extracts: characterisation, cell cytotoxicity, and its antifungal activity against *Candida albicans* in comparison to fluconazole. *IET Nanobiotechnology*.

[23] Seeley H. W., VanDemark P. J. (1962). *Microbes in Action. A Laboratory Manual of Microbiology*.

[24] Abdellatif A. A., Rasheed Z., Alhowail A. H. (2020). Silver citrate nanoparticles inhibit PMA-induced TNF*α* expression via deactivation of NF-*κ*B activity in human cancer cell-lines, MCF-7. *International Journal of Nanomedicine*.

[25] Do Q. D., Angkawijaya A. E., Tran-Nguyen P. L. (2014). Effect of extraction solvent on total phenol content, total flavonoid content, and antioxidant activity of *Limnophila aromatica*. *Journal of Food and Drug Analysis*.

[26] Prieto P., Pineda M., Aguilar M. (1999). Spectrophotometric quantitation of antioxidant capacity through the formation of a phosphomolybdenum complex: specific application to the determination of vitamin E. *Analytical Biochemistry*.

[27] Govindarajan R., Rastogi S., Vijayakumar M. (2003). Studies on the antioxidant activities of *Desmodium gangeticum*. *Biological and Pharmaceutical Bulletin*.

[28] Benzie I. F., Strain J. J. (1996). The ferric reducing ability of plasma (FRAP) as a measure of “antioxidant power”: the FRAP assay. *Analytical Biochemistry*.

[29] Mazor D., Greenberg L., Shamir D., Meyerstein D., Meyerstein N. (2006). Antioxidant properties of bucillamine: possible mode of action. *Biochemical and Biophysical Research Communications*.

[30] Birasuren B., Oh H. L., Kim C. R., Kim N. Y., Jeon H. L., Kim M. R. (2012). Antioxidant activities of *Ribes diacanthum* pall extracts in the northern region of Mongolia. *Preventive Nutrition and Food Science*.

[31] Wayne P. (2011). *Performance Standards for Antimicrobial Susceptibility Testing*.

[32] Abdallah E. M., Mujawah A. A. H., Al-Mijalli S. H. (2021). GC-MS and antibacterial potential of methanolic extract *Hyphaene thebaica* L. fruit pulp against antibiotics-resistant pathogens. *Journal of Pure and Applied Microbiology*.

[33] Gulluce M., Aslan A., Sokmen M. (2006). Screening the antioxidant and antimicrobial properties of the lichens *Parmelia saxatilis*, *Platismatia glauca*, *Ramalina pollinaria*, *Ramalina polymorpha* and *Umbilicaria nylanderiana*. *Phytomedicine*.

[34] Naomi L. (2008). *Nanosilver Toxicity: Ions, Nanoparticles or Both?*.

[35] Protima R., Siim K., Stanislav F., Erwan R. (2015). A review on the green synthesis of silver nanoparticles and their morphologies studied via TEM. *Advances in Materials Science and Engineering*.

[36] Kesharwani J., Yoon K. Y., Hwang J., Rai M. (2009). Phytofabrication of silver nanoparticles by leaf extract of *Datura metel*: hypothetical mechanism involved in synthesis. *Journal of Bionanoscience*.

[37] Kumar V., Gundampati R. K., Singh D. K., Jagannadham M. V., Sundar S., Hadi H. S. (2016). Photo-induced rapid biosynthesis of silver nanoparticle using aqueous extract of *Xanthium strumarium* and its antibacterial and antileishmanial activity. *Journal of Industrial and Engineering Chemistry*.

[38] Li S., Shen Y., Xie A. (2007). Green synthesis of silver nanoparticles using *Capsicum annuum* L. extract. *Journal of Green Chemistry*.

[39] Abdellatif A. A. H., Alturki H. N. H., Tawfeek H. M. (2021). Different cellulosic polymers for synthesizing silver nanoparticles with antioxidant and antibacterial activities. *Scientific Reports*.

[40] Abdellatif A. A. H., Mahmood A., Alsharidah M. (2022). Bioactivities of the green synthesized silver nanoparticles reduced using *Allium cepa* L aqueous extracts induced apoptosis in colorectal cancer cell lines. *Journal of Nanomaterials*.

[41] Abdellatif A. A. H., Sameh Tolba N., Al Rugaie O., Alhumaydhi F. A., Mousa A. M. (2022). Green synthesis of silver nanoparticles for enhancing wound healing activity in rats. *Saudi Pharmaceutical Journal*.

[42] Iravani S., Korbekandi H., Mirmohammadi S. V., Zolfaghari B. (2014). Synthesis of silver nanoparticles: chemical, physical and biological methods. *Research in Pharmaceutical Sciences*.

[43] Paramelle D., Sadovoy A., Gorelik S., Free P., Hobley J., Fernig D. G. (2014). A rapid method to estimate the concentration of citrate capped silver nanoparticles from UV-visible light spectra. *Analyst*.

[44] Sadeghi B., Gholamhoseinpoor F. (2015). A study on the stability and green synthesis of silver nanoparticles using *Ziziphora tenuior* (Zt) extract at room temperature. *Spectrochimica Acta. Part A, Molecular and Biomolecular Spectroscopy*.

[45] Helmlinger J., Sengstock C., Groß-Heitfeld C. (2016). Silver nanoparticles with different size and shape: equal cytotoxicity, but different antibacterial effects. *RSC Advances*.

[46] Gupta A., Koirala A. R., Gupta B., Parajuli N. (2019). Improved method for separation of silver nanoparticles synthesized using the *Nyctanthes arbor-tristis* shrub. *Acta Chemica Malaysia*.

[47] Zhang X. F., Liu Z. G., Shen W., Gurunathan S. (2016). Silver nanoparticles: synthesis, characterization, properties, applications, and therapeutic approaches. *International Journal of Molecular Sciences*.

[48] Abdellatif A. A. H., Khan R. A., Alhowail A. H. (2022). Octreotide-conjugated silver nanoparticles for active targeting of somatostatin receptors and their application in a nebulized rat model. *Nanotechnology Reviews*.

[49] Haggag E., Elshamy A., Rabeh M. (2019). Antiviral potential of green synthesized silver nanoparticles of *Lampranthus coccineus* and *Malephora lutea*. *International Journal of Nanomedicine*.

[50] Hamouda R. A., Hussein M. H., Abo-Elmagd R. A., Bawazir S. S. (2019). Synthesis and biological characterization of silver nanoparticles derived from the cyanobacterium *Oscillatoria limnetica*. *Scientific Reports*.

[51] Stojanovic R., Belscak-Cvitanovic A., Manojlovic V., Komes D., Nedovic V., Bugarski B. (2012). Encapsulation of Thyme (*Thymus serpyllum* L.) aqueous extract in calcium alginate beads. *Journal of the Science of Food and Agriculture*.

[52] Devi A., Das V. K., Deka D. (2017). Ginger extract as a nature based robust additive and its influence on the oxidation stability of biodiesel synthesized from non-edible oil. *Fuel*.

[53] Hseu Y.-C., Chang W.-H., Chen C.-S. (2008). Antioxidant activities of *Toona sinensis* leaves extracts using different antioxidant models. *Food and Chemical Toxicology*.

[54] Amarowicz R., Pegg R. B. (2019). Natural antioxidants of plant origin. *Advances in Food and Nutrition Research*.

[55] Drinka P., Niederman M. S., El-Solh A. A., Crnich C. J. (2011). Assessment of risk factors for multi-drug resistant organisms to guide empiric antibiotic selection in long term care: a dilemma. *Journal of the American Medical Directors Association*.

[56] Ruddaraju L. K., Pammi S. V. N., Guntuku G. S., Padavala V. S., Kolapalli V. R. M. (2020). A review on anti-bacterials to combat resistance: from ancient era of plants and metals to present and future perspectives of green nano technological combinations. *Asian Journal of Pharmaceutical Sciences*.

[57] Abdallah E. M. (2016). Antibacterial activity of *Hibiscus sabdariffa* L. calyces against hospital isolates of multidrug resistant *Acinetobacter baumannii*. *Journal of Acute Disease*.

[58] de Melo A. P. Z., de Oliveira Brisola Maciel M. V., Sganzerla W. G. (2020). Antibacterial activity, morphology, and physicochemical stability of biosynthesized silver nanoparticles using thyme (*Thymus vulgaris*) essential oil. *Materials Research Express*.

[59] Erci F., Cakir-Koc R., Isildak I. (2018). Green synthesis of silver nanoparticles using *Thymbra spicata* L. var. spicata (zahter) aqueous leaf extract and evaluation of their morphology-dependent antibacterial and cytotoxic activity. *Artificial Cells, Nanomedicine, and Biotechnology*.

[60] Mathew S., Prakash A., Radhakrishnan E. K. (2018). Sunlight mediated rapid synthesis of small size range silver nanoparticles using *Zingiber officinale* rhizome extract and its antibacterial activity analysis. *Inorganic and Nano-Metal Chemistry*.

[61] Abdallah E. M. (2011). Plants: an alternative source for antimicrobials. *Journal of Applied Pharmaceutical Science*.

[62] Kumar V., Yadav S. K. (2009). Plant-mediated synthesis of silver and gold nanoparticles and their applications. *Journal of Chemical Technology and Biotechnology*.

[63] Marambio‐Jones C., Hoek E. (2010). A review of the antibacterial effects of silver nanomaterials and potential implications for human health and the environment. *Journal of Nanoparticle Research*.

[64] Azócar M. I., Tamayo L., Vejar N. (2014). A systematic study of antibacterial silver nanoparticles: efficiency, enhanced permeability, and cytotoxic effects. *Journal of Nanoparticle Research*.

[65] Agnihotri S., Mukherji S., Mukherji S. (2013). Immobilized silver nanoparticles enhance contact killing and show highest efficacy: elucidation of the mechanism of bactericidal action of silver. *Nanoscale*.

